# (3*R*,4*R*,4a*S*,7a*R*,12b*S*)-3-Cyclo­propyl­methyl-4a,9-dihy­droxy-3-methyl-7-oxo-2,3,4,4a,5,6,7,7a-octa­hydro-1*H*-4,12-methano­benzofuro[3,2-*e*]isoquinolin-3-ium bromide

**DOI:** 10.1107/S1600536812000645

**Published:** 2012-01-14

**Authors:** Xiangfeng Chen, Zaiwei Zong, Youguo Du, Jianguo Li, Min Sun

**Affiliations:** a Nanjing Haiguang Applied Chemistry Institute, Jiangsu Aosaikang Pharmaceutical Co. Ltd, Nanjing 211112, People’s Republic of China; b School of Pharmacy, China Pharmaceutical University, Nanjing 210009, People’s Republic of China

## Abstract

The title compound, C_21_H_26_NO_4_^+^·Br^−^, also known as *R*-methyl­naltrexone (MNTX) bromide, is a selective peripher­ally acting μ-opioid receptor antagonist with a oroxymorphone skeleton, synthesized by hydroxyl protection, *N*-methyl­ation, deprotection and anion exchange of naltrexone. It comprises a five-ring system *A*/*B*/*C*/*D/E*. Rings *C* and *E* adopt distorted chair conformations, whereas ring *D* is in half-chair conformation. The *C*/*E* ring junctions are *trans* fused. The dihedral angle between rings *D* and *E* is 82.3 (1)°, while the dihedral angles between the planes of rings *C* and *A*, and rings *D* and *E* are respectively 81.7 (1), 75.9 (1) and 12.2 (1)°. In the crystal, mol­ecules are linked by O—H⋯Br hydrogen bonds.

## Related literature

For general background to methyl­naltrexone (MNTX) bromide, see: Garnock-Jones & McKeage (2010 ▶). For ring conformations, see: Cremer & Pople (1975 ▶). For synthesis of methyl­naltrexone bromide *via* hydroxyl protection, *N*-methyl­ation, deprotection and anion exchange, see: Doshan *et al.* (2010 ▶). 
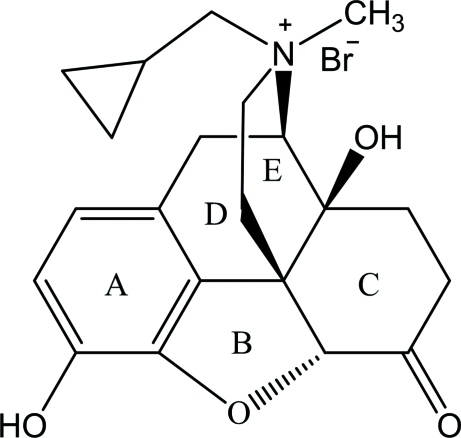


## Experimental

### 

#### Crystal data

C_21_H_26_NO_4_^+^·Br^−^
*M* *_r_* = 436.34
Monoclinic, 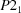

*a* = 7.708 (3) Å
*b* = 13.187 (5) Å
*c* = 9.501 (3) Å
β = 97.679 (6)°
*V* = 957.1 (6) Å^3^
*Z* = 2
Mo *K*α radiation
μ = 2.18 mm^−1^
*T* = 291 K
0.28 × 0.24 × 0.22 mm

#### Data collection

Bruker SMART APEX CCD diffractometer
Absorption correction: multi-scan (*SADABS*; Bruker, 2000 ▶) *T*_min_ = 0.581, *T*_max_ = 0.646
6955 measured reflections
4179 independent reflections
3259 reflections with *I* > 2σ(*I*)
*R*_int_ = 0.048

#### Refinement

*R*[*F* ^2^ > 2σ(*F* ^2^)] = 0.035
*wR*(*F* ^2^) = 0.073
*S* = 1.04
4179 reflections
245 parameters
2 restraints
H atoms treated by a mixture of independent and constrained refinement
Δρ_max_ = 0.39 e Å^−3^
Δρ_min_ = −0.44 e Å^−3^
Absolute structure: Flack (1983 ▶), 1636 Friedel pairs
Flack parameter: −0.006 (7)

### 

Data collection: *SMART* (Bruker, 2000 ▶); cell refinement: *SAINT* (Bruker, 2000 ▶); data reduction: *SAINT*; program(s) used to solve structure: *SHELXTL* (Sheldrick, 2008 ▶); program(s) used to refine structure: *SHELXTL*; molecular graphics: *SHELXTL*; software used to prepare material for publication: *SHELXTL* and *PLATON* (Spek, 2009 ▶).

## Supplementary Material

Crystal structure: contains datablock(s) I, global. DOI: 10.1107/S1600536812000645/bg2437sup1.cif

Structure factors: contains datablock(s) I. DOI: 10.1107/S1600536812000645/bg2437Isup2.hkl

Additional supplementary materials:  crystallographic information; 3D view; checkCIF report

## Figures and Tables

**Table 1:** Hydrogen-bond geometry (Å, °)

*D*—H⋯*A*	*D*—H	H⋯*A*	*D*⋯*A*	*D*—H⋯*A*
O1—H1*X*⋯Br1^i^	0.80 (5)	2.43 (5)	3.231 (3)	174 (5)
O4—H4*X*⋯Br1	0.81 (3)	2.40 (3)	3.204 (3)	174 (2)

Symmetry code: (i) 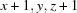.

**Fig. 1.**
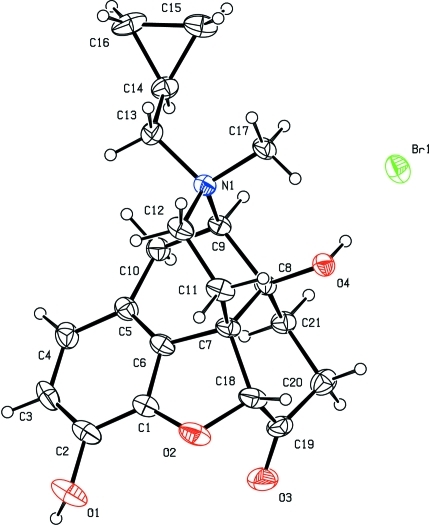
The molecular structure of the title compound with the atom-numbering scheme. Displacement ellipsoids are drawn at the 30% probability level.

## supplementary crystallographic information

### Comment

Methylnaltrexone bromide is a selective µ-opioid receptor antagonist that has restricted ability to cross the blood-brain barrier, thus enabling reversal of opioid induced peripheral effects, such as constipation, without affecting the central effects, such as pain relief (Garnock-Jones *et al.*, 2010).

The title compound was obtained by hydroxyl protection, *N*-methylation, deprotection and anion exchange of naltrexone. The molecule structure of (I) contains a five-ring system *A*/*B*/*C*/*D/E* (Fig. 1). Ring *A* is defined by atoms C1—C6, ring *B* by atoms C1/C6/C7/C18/O2, ring *C* by atoms C7/C8/C21/C20/C19/C18, ring *D* by atoms C5—C10, and ring *E* by atoms C9/C8/C7/C11/C12/N1. Ring *C* and *E* adopt a distorted chair conformation (Puckering parameters as defined by Cremer & Pople, 1975: *Q* = 0.558 (4)/ 0.604 (4) Å, *θ*= 158.7 (4)/ 13.4 (3) ° and *φ*= 334.6 (11)/ 145.3 (14) °, respectively), whereas ring *D* is in half chair conformation with puckering amplitude *Q* = 0.556 (4) Å, *θ*= 135.5 (4) ° *φ*=18.4 (5) °. The stereochemistry of the *C*/*E* ring junctions are *trans* fused. The dihedral angle between ring *D* and *E* is 82.3 (1)°, while the dihedral angles between the planes of ring *C* and the ring *A*, ring *D* and ring *E* are respectively 81.7 (1), 75.9 (1) and 12.2 (1)°. In the crystal structure, the molecules are linked by O—H···Br hydrogen bonds (Table 1).

### Experimental

To a solution of naltrexone (3.24 g, 9.5 mmol) in anhydrous tetrahydrofuran (THF) (200 mL) was added triethylamine (NEt_3_) (2.8 mL, 20 mmol) at 0 °C. After the reaction was stirred for 15 min at 0 °C, isobutyryl chloride (2.1 ml, 20 mmol) was added dropwise. Reaction mixture was stirred at 0 °C for 1 hr, then at room temperature for 18 hrs before being quenched with saturated sodium bicarbonate (NaHCO_3_) (aq) (140 ml) and 60 ml of H_2_O. The reaction was extracted with methylene chloride (CH_2_Cl_2_) (2×300 ml). The extracts were combined, washed with brine (200 ml), dried over sodium sulfate (Na_2_SO_4_) (50 g), filtered and concentrated *in vacuo*. The crude material was purified by flash chromatography on silica gel to give 3-O-isobutyryl-naltrexone (2.8 g 71.6%) as a white solid.

3-O-isobutyryl-naltrexone(1.4 g, 3.40 mmol) was transferred by spatula into a glass pressure vessel. The vessel was purged gently with nitrogen on the manifold for 5 minutes and was then evacuated under high vacuum. When the vacuum was constant, the lower part of the vessel was immersed in liquid nitrogen. Methyl iodide (2.0 g, 14.1 mmol) was dispensed into a separate flask on the manifold into a nitrogen atmosphere and frozen in liquid nitrogen. The frozen methyl iodide vessel was evacuated under high vacuum. The main manifold chamber was isolated from the high vacuum pump. The methyl iodide was allowed to warm to ambient temperature and sublime *via* the main chamber onto the liquid nitrogen cooled 3-O-isobutyryl-naltrexone. When sublimation was complete, nitrogen was slowly allowed to leach into the glass pressure vessel. The vessel was then sealed tight, removed from the manifold and heated in an oil bath at 88–90 °C for 17 hrs. The vessel was allowed to cool to ambient temperature before allowing nitrogen to flow into the vessel. The vessel was then evacuated under high vacuum to remove residues of unreacted methyl iodide giving a white solid. The crude material was purified by flash chromatography on silica gel to give 3-*O*-isobutyryl-*N*-methylnaltrexone iodide salt (1.68 g, 89.3%) as a white solid.

3-*O*-isobutyryl-*N*-methylnaltrexone iodide salt (1.68 g, 3.04 mmol) was dissolved in methanol (25 ml). To this mixture was added sterile water (23 ml) followed by 48% aqueous hydrobromic acid (3 ml). The resultant mixture was stirred under nitrogen and heated in an oil bath at 64–65 °C for 6.5 hrs. The mixture was concentrated on the rotary evaporator with the bath at 22–25 °C until approximately 1 ml of oily liquid remained. Acetonitrile (20 ml) was added and the mixture was reconcentrated. This was repeated a further three times, using 20 ml of acetonitrile, to give a ginger colored crisp foam (1.1 g, 83% crude yield).

The foam (1.1 g) was dispersed in water (12 ml)/methanol (4 ml). Some dark oil remained undissolved. The clear supernatant liquid was decanted and applied to the prepared anion exchange resin column. The residue was washed twice with methanol (0.4 mL)/water (6 ml). The supernatant liquors were applied to the column. The colunm was eluted with 4.2 *L* of sterile water and fractions of ~20 ml were collected. The yield of the white solid *N*-methylnaltrexone bromide 814.4 mg (64%).

### Refinement

The H atom of the hydroxy groups were located from the difference map and refined isotropically. The remaining H atoms were placed in geometrical positions and constrained to ride on their parent atoms with C–H distances in the range 0.93–0.98 Å, and refined with *U*_iso_(H) = 1.5*U*_eq_(C) for methyl H and 1.2*U*_eq_(C) for other H atoms.

### Crystal data

**Table:**

C_21_H_26_NO_4_^+^·Br^−^	*F*(000) = 452
*M**_r_* = 436.34	*D*_x_ = 1.514 Mg m^−^^3^
Monoclinic, *P*2_1_	Mo *K*α radiation, λ = 0.71073 Å
Hall symbol: P 2yb	Cell parameters from 2706 reflections
*a* = 7.708 (3) Å	θ = 2.3–24.6°
*b* = 13.187 (5) Å	µ = 2.18 mm^−^^1^
*c* = 9.501 (3) Å	*T* = 291 K
β = 97.679 (6)°	Block, colourless
*V* = 957.1 (6) Å^3^	0.28 × 0.24 × 0.22 mm
*Z* = 2	

### Data collection

**Table:**

Bruker SMART APEX CCD diffractometer	4179 independent reflections
Radiation source: sealed tube	3259 reflections with *I* > 2σ(*I*)
graphite	*R*_int_ = 0.048
phi and ω scans	θ_max_ = 28.5°, θ_min_ = 2.2°
Absorption correction: multi-scan (*SADABS*; Bruker, 2000)	*h* = −7→10
*T*_min_ = 0.581, *T*_max_ = 0.646	*k* = −12→17
6955 measured reflections	*l* = −12→11

### Refinement

**Table:**

Refinement on *F*^2^	Secondary atom site location: difference Fourier map
Least-squares matrix: full	Hydrogen site location: inferred from neighbouring sites
*R*[*F*^2^ > 2σ(*F*^2^)] = 0.035	H atoms treated by a mixture of independent and constrained refinement
*wR*(*F*^2^) = 0.073	*w* = 1/[σ^2^(*F*_o_^2^) + (0.020*P*)^2^] where *P* = (*F*_o_^2^ + 2*F*_c_^2^)/3
*S* = 1.04	(Δ/σ)_max_ = 0.001
4179 reflections	Δρ_max_ = 0.39 e Å^−^^3^
245 parameters	Δρ_min_ = −0.44 e Å^−^^3^
2 restraints	Absolute structure: Flack (1983), 1636 Friedel pairs
Primary atom site location: structure-invariant direct methods	Flack parameter: −0.006 (7)

### Special details

**Table:**

Geometry. All esds (except the esd in the dihedral angle between two l.s. planes) are estimated using the full covariance matrix. The cell esds are taken into account individually in the estimation of esds in distances, angles and torsion angles; correlations between esds in cell parameters are only used when they are defined by crystal symmetry. An approximate (isotropic) treatment of cell esds is used for estimating esds involving l.s. planes.
Refinement. Refinement of *F*^2^ against ALL reflections. The weighted *R*-factor *wR* and goodness of fit *S* are based on *F*^2^, conventional *R*-factors *R* are based on *F*, with *F* set to zero for negative *F*^2^. The threshold expression of *F*^2^ > σ(*F*^2^) is used only for calculating *R*-factors(gt) *etc*. and is not relevant to the choice of reflections for refinement. *R*-factors based on *F*^2^ are statistically about twice as large as those based on *F*, and *R*- factors based on ALL data will be even larger.

### Fractional atomic coordinates and isotropic or equivalent isotropic displacement parameters (Å^2^)

**Table:**

	*x*	*y*	*z*	*U*_iso_*/*U*_eq_	
Br1	−0.27040 (4)	0.31792 (5)	0.03917 (3)	0.05094 (11)	
C1	0.5410 (4)	0.4248 (3)	0.5393 (4)	0.0436 (9)	
C2	0.5975 (4)	0.4559 (3)	0.6764 (4)	0.0506 (10)	
C3	0.4825 (5)	0.5137 (3)	0.7423 (4)	0.0519 (10)	
H3	0.5192	0.5375	0.8336	0.062*	
C4	0.3139 (5)	0.5383 (3)	0.6787 (3)	0.0464 (9)	
H4	0.2389	0.5748	0.7287	0.056*	
C5	0.2590 (4)	0.5076 (3)	0.5394 (3)	0.0392 (8)	
C6	0.3776 (4)	0.4540 (3)	0.4748 (3)	0.0372 (8)	
C7	0.3529 (4)	0.4120 (3)	0.3254 (3)	0.0358 (8)	
C8	0.1586 (4)	0.3871 (3)	0.2814 (3)	0.0330 (7)	
C9	0.0565 (4)	0.4882 (3)	0.3026 (3)	0.0320 (7)	
H9	−0.0676	0.4745	0.2710	0.038*	
C10	0.0725 (4)	0.5145 (2)	0.4634 (3)	0.0405 (8)	
H10A	0.0292	0.5828	0.4737	0.049*	
H10B	−0.0013	0.4687	0.5088	0.049*	
C11	0.4099 (4)	0.4890 (3)	0.2210 (3)	0.0425 (9)	
H11A	0.5342	0.5021	0.2444	0.051*	
H11B	0.3901	0.4611	0.1257	0.051*	
C12	0.3096 (4)	0.5877 (3)	0.2249 (3)	0.0398 (8)	
H12A	0.3397	0.6316	0.1499	0.048*	
H12B	0.3468	0.6213	0.3147	0.048*	
C13	0.0381 (4)	0.67932 (19)	0.2407 (3)	0.0406 (8)	
H13A	0.0929	0.7008	0.3338	0.049*	
H13B	0.0694	0.7280	0.1719	0.049*	
C14	−0.1571 (4)	0.6804 (3)	0.2385 (3)	0.0432 (8)	
H14	−0.2058	0.6347	0.3045	0.052*	
C15	−0.2726 (5)	0.7068 (3)	0.1044 (4)	0.0565 (11)	
H15A	−0.2176	0.7162	0.0192	0.068*	
H15B	−0.3881	0.6764	0.0887	0.068*	
C16	−0.2390 (5)	0.7841 (3)	0.2174 (4)	0.058	
H16A	−0.3338	0.8006	0.2710	0.070*	
H16B	−0.1632	0.8405	0.2015	0.070*	
C17	0.0470 (4)	0.5559 (3)	0.0514 (3)	0.0395 (8)	
H17A	0.0618	0.6163	−0.0020	0.059*	
H17B	−0.0747	0.5377	0.0404	0.059*	
H17C	0.1132	0.5016	0.0173	0.059*	
C18	0.4825 (3)	0.3216 (4)	0.3524 (3)	0.0451 (7)	
H18	0.5266	0.3014	0.2645	0.054*	
C19	0.3966 (5)	0.2325 (3)	0.4176 (4)	0.0465 (9)	
C20	0.2143 (5)	0.2074 (3)	0.3494 (4)	0.0488 (9)	
H20A	0.1708	0.1484	0.3946	0.059*	
H20B	0.2141	0.1927	0.2494	0.059*	
C21	0.0982 (4)	0.2997 (3)	0.3677 (3)	0.0398 (9)	
H21A	−0.0232	0.2836	0.3347	0.048*	
H21B	0.1078	0.3184	0.4671	0.048*	
N1	0.1112 (3)	0.5747 (2)	0.2073 (3)	0.0335 (6)	
O1	0.7622 (3)	0.4286 (3)	0.7404 (3)	0.0719 (10)	
H1X	0.762 (6)	0.403 (4)	0.817 (5)	0.086*	
O2	0.6235 (3)	0.3609 (2)	0.4553 (2)	0.0548 (8)	
O3	0.4644 (4)	0.1902 (2)	0.5242 (3)	0.0638 (9)	
O4	0.1381 (3)	0.35700 (17)	0.1374 (2)	0.0393 (6)	
H4X	0.037 (4)	0.344 (3)	0.108 (3)	0.047*	

### Atomic displacement parameters (Å^2^)

**Table:**

	*U*^11^	*U*^22^	*U*^33^	*U*^12^	*U*^13^	*U*^23^
Br1	0.04321 (15)	0.0671 (2)	0.04230 (17)	−0.0020 (2)	0.00507 (11)	−0.0091 (2)
C1	0.0374 (18)	0.051 (3)	0.040 (2)	0.0021 (17)	−0.0021 (15)	0.0060 (17)
C2	0.0402 (18)	0.064 (3)	0.044 (2)	−0.0095 (18)	−0.0098 (15)	0.0156 (18)
C3	0.061 (2)	0.061 (3)	0.0297 (18)	−0.021 (2)	−0.0071 (16)	0.0019 (17)
C4	0.061 (2)	0.046 (2)	0.0322 (18)	−0.0040 (18)	0.0057 (15)	0.0002 (16)
C5	0.0437 (18)	0.044 (2)	0.0299 (16)	−0.0031 (16)	0.0036 (13)	0.0046 (15)
C6	0.0340 (17)	0.048 (2)	0.0286 (17)	−0.0016 (16)	0.0022 (13)	0.0085 (16)
C7	0.0337 (16)	0.046 (2)	0.0284 (16)	0.0083 (14)	0.0059 (12)	0.0059 (15)
C8	0.0318 (15)	0.041 (2)	0.0260 (15)	0.0057 (14)	0.0041 (12)	0.0001 (14)
C9	0.0282 (14)	0.040 (2)	0.0283 (15)	0.0008 (13)	0.0045 (12)	0.0048 (14)
C10	0.0440 (18)	0.048 (2)	0.0305 (16)	0.0079 (16)	0.0079 (13)	−0.0009 (15)
C11	0.0295 (15)	0.058 (3)	0.0409 (18)	0.0000 (16)	0.0082 (13)	0.0117 (17)
C12	0.0274 (15)	0.050 (2)	0.0413 (18)	−0.0081 (15)	0.0020 (13)	0.0094 (16)
C13	0.0469 (18)	0.033 (2)	0.0417 (17)	0.0000 (16)	0.0036 (14)	−0.0012 (15)
C14	0.0475 (19)	0.042 (2)	0.0420 (18)	0.0075 (16)	0.0127 (15)	0.0091 (16)
C15	0.050 (2)	0.072 (3)	0.047 (2)	0.011 (2)	0.0065 (16)	0.022 (2)
C16	0.062	0.052	0.066	0.029	0.031	0.019
C17	0.0460 (19)	0.045 (2)	0.0257 (16)	0.0010 (16)	−0.0012 (13)	0.0055 (15)
C18	0.0359 (14)	0.064 (2)	0.0348 (14)	0.016 (2)	0.0037 (11)	0.008 (2)
C19	0.056 (2)	0.048 (3)	0.0360 (19)	0.0243 (19)	0.0088 (16)	0.0002 (18)
C20	0.062 (2)	0.039 (2)	0.047 (2)	0.0038 (19)	0.0162 (18)	0.0040 (17)
C21	0.0403 (15)	0.042 (3)	0.0380 (15)	0.0039 (16)	0.0074 (12)	0.0049 (16)
N1	0.0306 (12)	0.0356 (17)	0.0337 (14)	−0.0007 (12)	0.0020 (10)	0.0028 (12)
O1	0.0516 (15)	0.105 (3)	0.0531 (16)	−0.0082 (16)	−0.0165 (13)	0.0250 (17)
O2	0.0337 (11)	0.083 (2)	0.0459 (13)	0.0126 (12)	−0.0002 (10)	0.0088 (12)
O3	0.081 (2)	0.059 (2)	0.0489 (16)	0.0221 (18)	−0.0009 (15)	0.0115 (15)
O4	0.0431 (11)	0.0443 (17)	0.0300 (11)	0.0049 (10)	0.0029 (9)	−0.0029 (9)

### Geometric parameters (Å, °)

**Table:**

C1—O2	1.374 (4)	C13—C14	1.502 (5)
C1—C2	1.379 (5)	C13—N1	1.539 (4)
C1—C6	1.380 (4)	C13—H13A	0.9700
C2—O1	1.380 (4)	C13—H13B	0.9700
C2—C3	1.381 (5)	C14—C15	1.495 (4)
C3—C4	1.396 (5)	C14—C16	1.508 (5)
C3—H3	0.9300	C14—H14	0.9800
C4—C5	1.395 (4)	C15—C16	1.478 (6)
C4—H4	0.9300	C15—H15A	0.9700
C5—C6	1.364 (5)	C15—H15B	0.9700
C5—C10	1.524 (4)	C16—H16A	0.9700
C6—C7	1.512 (4)	C16—H16B	0.9700
C7—C11	1.524 (5)	C17—N1	1.518 (4)
C7—C8	1.536 (4)	C17—H17A	0.9600
C7—C18	1.555 (5)	C17—H17B	0.9600
C8—O4	1.413 (3)	C17—H17C	0.9600
C8—C21	1.523 (5)	C18—O2	1.457 (4)
C8—C9	1.575 (4)	C18—C19	1.520 (6)
C9—N1	1.549 (4)	C18—H18	0.9800
C9—C10	1.555 (4)	C19—O3	1.212 (4)
C9—H9	0.9800	C19—C20	1.504 (5)
C10—H10A	0.9700	C20—C21	1.534 (5)
C10—H10B	0.9700	C20—H20A	0.9700
C11—C12	1.517 (5)	C20—H20B	0.9700
C11—H11A	0.9700	C21—H21A	0.9700
C11—H11B	0.9700	C21—H21B	0.9700
C12—N1	1.526 (4)	O1—H1X	0.80 (5)
C12—H12A	0.9700	O4—H4X	0.82 (3)
C12—H12B	0.9700		
			
O2—C1—C2	128.2 (3)	C14—C13—H13B	108.9
O2—C1—C6	112.3 (3)	N1—C13—H13B	108.9
C2—C1—C6	119.4 (4)	H13A—C13—H13B	107.7
C1—C2—O1	119.6 (4)	C15—C14—C13	119.7 (3)
C1—C2—C3	116.8 (3)	C15—C14—C16	59.0 (2)
O1—C2—C3	123.6 (3)	C13—C14—C16	114.3 (3)
C2—C3—C4	123.3 (3)	C15—C14—H14	117.0
C2—C3—H3	118.4	C13—C14—H14	117.0
C4—C3—H3	118.4	C16—C14—H14	117.0
C5—C4—C3	119.3 (3)	C16—C15—C14	61.0 (2)
C5—C4—H4	120.3	C16—C15—H15A	117.7
C3—C4—H4	120.3	C14—C15—H15A	117.7
C6—C5—C4	116.2 (3)	C16—C15—H15B	117.7
C6—C5—C10	117.7 (3)	C14—C15—H15B	117.7
C4—C5—C10	125.4 (3)	H15A—C15—H15B	114.8
C5—C6—C1	124.8 (3)	C15—C16—C14	60.1 (2)
C5—C6—C7	127.2 (3)	C15—C16—H16A	117.8
C1—C6—C7	107.9 (3)	C14—C16—H16A	117.8
C6—C7—C11	110.9 (3)	C15—C16—H16B	117.8
C6—C7—C8	109.2 (2)	C14—C16—H16B	117.8
C11—C7—C8	108.7 (2)	H16A—C16—H16B	114.9
C6—C7—C18	97.4 (2)	N1—C17—H17A	109.5
C11—C7—C18	112.5 (3)	N1—C17—H17B	109.5
C8—C7—C18	117.5 (3)	H17A—C17—H17B	109.5
O4—C8—C21	107.8 (3)	N1—C17—H17C	109.5
O4—C8—C7	107.6 (2)	H17A—C17—H17C	109.5
C21—C8—C7	111.9 (2)	H17B—C17—H17C	109.5
O4—C8—C9	111.6 (2)	O2—C18—C19	109.1 (2)
C21—C8—C9	112.2 (2)	O2—C18—C7	104.1 (4)
C7—C8—C9	105.6 (3)	C19—C18—C7	110.8 (2)
N1—C9—C10	114.7 (3)	O2—C18—H18	110.9
N1—C9—C8	111.7 (2)	C19—C18—H18	110.9
C10—C9—C8	109.8 (2)	C7—C18—H18	110.9
N1—C9—H9	106.7	O3—C19—C20	122.1 (4)
C10—C9—H9	106.7	O3—C19—C18	122.3 (3)
C8—C9—H9	106.7	C20—C19—C18	115.3 (3)
C5—C10—C9	113.5 (2)	C19—C20—C21	107.6 (3)
C5—C10—H10A	108.9	C19—C20—H20A	110.2
C9—C10—H10A	108.9	C21—C20—H20A	110.2
C5—C10—H10B	108.9	C19—C20—H20B	110.2
C9—C10—H10B	108.9	C21—C20—H20B	110.2
H10A—C10—H10B	107.7	H20A—C20—H20B	108.5
C12—C11—C7	111.3 (3)	C8—C21—C20	108.3 (3)
C12—C11—H11A	109.4	C8—C21—H21A	110.0
C7—C11—H11A	109.4	C20—C21—H21A	110.0
C12—C11—H11B	109.4	C8—C21—H21B	110.0
C7—C11—H11B	109.4	C20—C21—H21B	110.0
H11A—C11—H11B	108.0	H21A—C21—H21B	108.4
C11—C12—N1	114.0 (3)	C17—N1—C12	108.4 (2)
C11—C12—H12A	108.7	C17—N1—C13	105.5 (2)
N1—C12—H12A	108.7	C12—N1—C13	105.4 (2)
C11—C12—H12B	108.7	C17—N1—C9	111.8 (2)
N1—C12—H12B	108.7	C12—N1—C9	111.5 (2)
H12A—C12—H12B	107.6	C13—N1—C9	113.8 (2)
C14—C13—N1	113.5 (3)	C2—O1—H1X	114 (3)
C14—C13—H13A	108.9	C1—O2—C18	104.3 (2)
N1—C13—H13A	108.9	C8—O4—H4X	112 (2)
			
O2—C1—C2—O1	5.5 (6)	C8—C9—C10—C5	47.6 (3)
C6—C1—C2—O1	−178.7 (4)	C6—C7—C11—C12	−57.6 (3)
O2—C1—C2—C3	−174.4 (4)	C8—C7—C11—C12	62.5 (3)
C6—C1—C2—C3	1.3 (6)	C18—C7—C11—C12	−165.5 (3)
C1—C2—C3—C4	2.1 (6)	C7—C11—C12—N1	−52.0 (4)
O1—C2—C3—C4	−177.8 (4)	N1—C13—C14—C15	−92.0 (4)
C2—C3—C4—C5	−3.0 (6)	N1—C13—C14—C16	−158.8 (2)
C3—C4—C5—C6	0.3 (5)	C13—C14—C15—C16	−102.1 (4)
C3—C4—C5—C10	170.4 (3)	C13—C14—C16—C15	111.3 (3)
C4—C5—C6—C1	3.3 (6)	C6—C7—C18—O2	−35.8 (3)
C10—C5—C6—C1	−167.6 (4)	C11—C7—C18—O2	80.5 (3)
C4—C5—C6—C7	−179.8 (3)	C8—C7—C18—O2	−152.0 (2)
C10—C5—C6—C7	9.3 (5)	C6—C7—C18—C19	81.4 (3)
O2—C1—C6—C5	172.2 (4)	C11—C7—C18—C19	−162.3 (3)
C2—C1—C6—C5	−4.2 (6)	C8—C7—C18—C19	−34.8 (4)
O2—C1—C6—C7	−5.2 (4)	O2—C18—C19—O3	−14.9 (5)
C2—C1—C6—C7	178.4 (3)	C7—C18—C19—O3	−129.0 (3)
C5—C6—C7—C11	90.1 (4)	O2—C18—C19—C20	159.1 (3)
C1—C6—C7—C11	−92.6 (4)	C7—C18—C19—C20	45.1 (4)
C5—C6—C7—C8	−29.7 (5)	O3—C19—C20—C21	112.0 (4)
C1—C6—C7—C8	147.6 (3)	C18—C19—C20—C21	−62.0 (4)
C5—C6—C7—C18	−152.3 (4)	O4—C8—C21—C20	61.1 (3)
C1—C6—C7—C18	25.1 (4)	C7—C8—C21—C20	−57.1 (3)
C6—C7—C8—O4	174.7 (3)	C9—C8—C21—C20	−175.6 (2)
C11—C7—C8—O4	53.6 (4)	C19—C20—C21—C8	66.2 (3)
C18—C7—C8—O4	−75.7 (3)	C11—C12—N1—C17	−77.1 (3)
C6—C7—C8—C21	−67.0 (4)	C11—C12—N1—C13	170.2 (2)
C11—C7—C8—C21	171.8 (3)	C11—C12—N1—C9	46.3 (3)
C18—C7—C8—C21	42.6 (3)	C14—C13—N1—C17	69.2 (3)
C6—C7—C8—C9	55.4 (3)	C14—C13—N1—C12	−176.2 (2)
C11—C7—C8—C9	−65.7 (3)	C14—C13—N1—C9	−53.7 (3)
C18—C7—C8—C9	165.0 (2)	C10—C9—N1—C17	−164.4 (2)
O4—C8—C9—N1	−55.3 (3)	C8—C9—N1—C17	69.9 (3)
C21—C8—C9—N1	−176.5 (2)	C10—C9—N1—C12	74.1 (3)
C7—C8—C9—N1	61.3 (3)	C8—C9—N1—C12	−51.7 (3)
O4—C8—C9—C10	176.3 (2)	C10—C9—N1—C13	−45.0 (3)
C21—C8—C9—C10	55.1 (3)	C8—C9—N1—C13	−170.7 (2)
C7—C8—C9—C10	−67.1 (3)	C2—C1—O2—C18	156.9 (4)
C6—C5—C10—C9	−18.0 (4)	C6—C1—O2—C18	−19.2 (4)
C4—C5—C10—C9	172.1 (3)	C19—C18—O2—C1	−83.5 (3)
N1—C9—C10—C5	−79.1 (3)	C7—C18—O2—C1	34.8 (3)

### Hydrogen-bond geometry (°)

**Table:**

*D*—H···*A*
—···
—···
